# Assessment of strategic healthcare purchasing and financial autonomy in Tanzania: the case of results-based financing and health basket fund

**DOI:** 10.3389/fpubh.2023.1260236

**Published:** 2024-01-12

**Authors:** John Maiba, Neha S. Singh, Rachel Cassidy, Gemini Mtei, Josephine Borghi, Ntuli A. Kapologwe, Peter Binyaruka

**Affiliations:** ^1^Department of Health System, Impact Evaluation, and Policy, Ifakara Health Institute, Dar es Salaam, Tanzania; ^2^Department of Global Health and Development, London School of Hygiene and Tropical Medicine, London, United Kingdom; ^3^Geneva Centre of Humanitarian Studies, University of Geneva, Geneva, Switzerland; ^4^Abt Associates Inc., USAID Public Sector Systems Strengthening (PS3+) Project, Dar es Salaam, Tanzania; ^5^President's Office, Regional Administration, and Local Government Tanzania – (PO-RALG), Dodoma, Tanzania

**Keywords:** strategic purchasing, contracting, payment method, autonomy, Tanzania

## Abstract

**Background:**

Low-and middle-income countries (LMICs) are implementing health financing reforms toward Universal Health Coverage (UHC). In Tanzania direct health facility financing of health basket funds (DHFF-HBF) scheme was introduced in 2017/18, while the results-based financing (RBF) scheme was introduced in 2016. The DHFF-HBF involves a direct transfer of pooled donor funds (Health Basket Funds, HBF) from the central government to public primary healthcare-PHC (including a few selected non-public PHC with a service agreement) facilities bank accounts, while the RBF involves paying providers based on pre-defined performance indicators or targets in PHC facilities. We consider whether these two reforms align with strategic healthcare purchasing principles by describing and comparing their purchasing arrangements and associated financial autonomy.

**Methods:**

We used document review and qualitative methods. Key policy documents and articles related to strategic purchasing and financial autonomy were reviewed. In-depth interviews were conducted with health managers and providers (*n* = 31) from 25 public facilities, health managers (*n* = 4) in the Mwanza region (implementing DHFF-HBF and RBF), and national-level stakeholders (*n* = 2). In this paper, we describe and compare DHFF-HBF and RBF in terms of four functions of strategic purchasing (benefit specification, contracting, payment method, and performance monitoring), but also compare the degree of purchaser-provider split and financial autonomy. Interviews were recorded, transcribed verbatim, and analyzed using a thematic framework approach.

**Results:**

The RBF paid facilities based on 17 health services and 18 groups of quality indicators, whilst the DHFF-HBF payment accounts for performance on two quality indicators, six service indicators, distance from district headquarters, and population catchment size. Both schemes purchased services from PHC facilities (dispensaries, health centers, and district hospitals). RBF uses a fee-for-service payment adjusted by the quality of care score method adjusted by quality of care score, while the DHFF-HBF scheme uses a formula-based capitation payment method with adjustors. Unlike DHFF-HBF which relies on an annual general auditing process, the RBF involved more detailed and intensive performance monitoring including data before verification prior to payment across all facilities on a quarterly basis. RBF scheme had a clear purchaser-provider split arrangement compared to a partial arrangement under the DHFF-HBF scheme. Study participants reported that the RBF scheme provided more autonomy on spending facility funds, while the DHFF-HBF scheme was less flexible due to a budget ceiling on specific spending items.

**Conclusion:**

Both RBF and DHFF-HBF considered most of the strategic healthcare purchasing principles, but further efforts are needed to strengthen the alignment towards UHC. This may include further strengthening the data verification process and spending autonomy for DHFF-HBF, although it is important to contain costs associated with verification and ensuring public financial management around spending autonomy.

## 1 Background

Many governments around the world are implementing health financing reforms to move towards Universal Health Coverage (UHC). The aim of these reforms is to design health financing systems that raise sufficient funds and to ensure that every citizen has access to quality healthcare without financial hardship due to healthcare payment (1). Most government efforts were initially directed at resource generation with fewer efforts on how to purchase health services from health providers using pooled resources (2–4). However, recently the importance of strategic health purchasing (SHP) has been recognized, whereby payments are based on outputs in contrast to traditional input-based payments to accelerate progress toward UHC (2, 5). SHP encompasses questions around: what to buy (benefit package), from whom to buy (providers), and how to contract and pay (3, 5, 6), and is relevant to funds from the government, insurance, or donors. SHP is increasingly recognized as an important tool to enhance the use and equitable allocation of resources, which is especially important in resource-constrained health systems in low and middle-income countries (LMICs) (2, 6, 7). SHP could potentially enhance provider responsiveness and efficiency toward enhancing overall health system performance (8–10).

For healthcare purchasing to be strategic both the purchasers and providers (health facilities) should have a clear decision-making space as well as a sufficient level of autonomy in determining how funds are used (11). Financial autonomy especially among health providers is critical such that a health facility has the status to receive, manage, and account for funds from any legal source or funds flow (10), typically through opening facility bank accounts and including facilities in the country’s chart of accounts as spending entities (1). Promoting facility financial autonomy to meet critical priorities, such as the management of funds and the right to determine and procure the best mix of inputs, is especially important in the context of limited resources.

In sub-Saharan African (SSA) countries, there is a growing interest in assessing SHP arrangements in health financing and the degree to which they have enhanced progress towards UHC and offer autonomy to providers. However, much of the existing research has focused on health insurance [in Kenya (12) and Tanzania (9)], and government purchasing arrangements [in Nigeria (13) and Uganda (14)]. However, there is only one study in SSA that assessed the SHP of donor-funding mechanisms and compared different donor-funded purchasing arrangements in Uganda (14). To our knowledge, there is no study that has assessed the degree of financial autonomy given to providers by different purchasing arrangements.

Over the last decades, donors and development partners have promoted the use of Result Based Financing (RBF) as a way to channel funding directly to front-line facilities based on their performance (15). The RBF funding is tied to specific outputs and quality measures, and so typically has tighter systems of verification (16). However, there is growing move of implementing mechanisms of financing facilities directly such as direct health facility financing (DHFF) (10, 16). DHFF involves providing financial resources directly to primary healthcare (PHC) facilities’ bank accounts to meet various facility needs (10). This may include direct transfer of decentralized operating grants (17) and/or pooled donor funds (18–20). Both schemes have the potential of improving service utilization and delivery in PHC facilities. In Tanzania, for example, both schemes associated with an increase in the availability of drugs and supplies, enhanced provider autonomy (budgeting and spending), and accountability through the health facility governing committee (HFGC) (21). In Nigeria, both DFF and RBF programs improved healthcare quality, including drug availability, equipment, hygiene facilities, waste management, and increased outreach efforts (17). Recent research also pointed to the costs of the RBF scheme being nearly twice as expensive as the DHFF scheme (22), with little difference in outcomes (23). Higher costs for the RBF scheme driven much by costs associated with providers incentive payments, data verification activities, and administration or operation cost (24–28).

Although both schemes, RBF and DHFF, purchase health services strategically, there is no comparison to date between these schemes in terms of SHP arrangements and financial autonomy. This study aimed to fill this knowledge gap by using two schemes of transferring donor funds directly to public PHC facilities (including few nonpublic PHC with a service agreement) in Tanzania – RBF and DHFF. The findings from this assessment provides evidence on how the two schemes incorporated SHP functions and financial autonomy, which can inform policy makers when design facility financing mechanisms to promote efficiency in resource allocation and spending towards UHC.

## 2 Methods

### 2.1 Study setting

The study was conducted in Tanzania, a lower middle-income country in East Africa. Tanzania’s health system is funded through multiple sources including the government through general taxation (22%), donor support (34%), out-of-pocket payments (32%), and health insurance contributions (12%) (29). The Tanzania health financing strategy (2016–2026) (30) emphasizes the need to ensure SHP for UHC and overall health system performance, which promoted the introduction of financing reforms on funding facilities strategically.

### 2.2 The RBF scheme in Tanzania

The RBF scheme was implemented in nine regions (Mwanza, Pwani, Shinyanga, Geita, Kigoma, Kagera, Mara, Simiyu, and Tabora) (2016–2020), and was funded by the World Bank and the USAID (Table 1) (21, 31). The scheme was designed to improve health service use and equity, as well as the quality and efficiency of health care, particularly among public PHC (including a few selected nonpublic PHC with a service agreement) facilities (31). The RBF scheme paid health providers (facility and/or health workers) based on pre-defined performance indicators including 17 quantitative indicators of service utilization and 18 groups of quality items. One of the eligibility criteria for a public PHC facility to implement the RBF scheme was to have at least one star, following a star rating assessment that was done to assess the level of structural quality of care across facilities (32). Investment or startup grant of TZS 10 million was provided to selected facilities to facilitate scheme initiation, to promote innovation and development of new approaches and solutions (21). RBF implementers were trained using a training of trainer’s approach in cascade. The national RBF team trained regional and district health managers, who, in turn, trained healthcare providers in their districts. The RBF training at the district level included representatives from dispensaries (*n* = 2), health centers (*n* = 5), hospitals (*n* = 10), HFGC (*n* = 2), and Community Health Workers (CHWs) (*n* = 2) (31). RBF payments were then made on a quarterly basis after data verification. The payments were split between bonuses to staff members (maximum 25%) and the remaining amount of investment funds for facility operations or demand creation initiatives. The RBF schemes set maximum funding ceiling, varying based on the type of health facility: dispensaries capped at (TZS 4,961,674), health centers (TZS 19,900,459), and hospitals (TZS 71,405,166) adjusted based on equity considerations (31). The evaluation of RBF showed improvements in some incentivized indicators and other process outcomes despite some implementation challenges such as payment delays (21).

### 2.3 The DHFF scheme in Tanzania

In Tanzania, DHFF started with the direct transfer of “health basket funds” (HBF), as pooled donor funds earmarked for the health sector, from central Ministry of Finance to public PHC (including few nonpublic PHC with a service agreement) facilities, here referred to as DHFF-HBF. The DHFF-HBF scheme is implemented in all public PHC facilities across all districts in Tanzania since 2017/2018 (18, 33, 34). Prior to DHFF scheme, the HBF was directly transferred from central government to district councils, whereby councils were responsible for controlling, planning and budgeting for facility level activities (18). Funding PHC facilities through district councils was deemed bureaucratic with chronic delays in allocating funds to facilities in order to meet various need (35). The DHFF-HBF approach enables public PHC facilities to receive funds directly into their bank account and manage them independently to meet the needs of the population (10). This approach is designed to ensure reliable and timely disbursement of funds, better-matching payment to local priorities, and enhancement of autonomy, transparency, and accountability at the PHC level (18, 36). The training of trainer’s approach was used, whereby the training started with national stakeholders and regional managers, who then trained district managers and accountants, representatives from PHC facilities including district hospitals (10), health centers (*n* = 2) and dispensary (*n* = 1) (37). The scheme sets specific expenditure ceilings for providers, allocating funds to different areas including 35% for health commodities, 15% for strengthen human resources for health management capacity, 45% for reproductive, maternal, newborn, child, and adolescent health (RMNCAH), and 5% for community health system (37). These allocation percentages aim to guide and prioritize resource allocation within the healthcare facilities.

**Table 1:** Comparison table between RBF and DHFF-HBF in Tanzania.

Scheme attributes	RBF	DHFF-HBF
Funders	World Bank & USAID	Development partners through a health basket pool of funds to strengthening health system
Piloting & early initiations	Pay-for-performance (P4P) pilot in Pwani region (2011–2014), followed by RBF pilot in Shinyanga region (2015)	The government and HBF partners signed MoU of adopting DHFF in 2015. Actual DHFF implementation started in December 2017
Facilitator (makes follow ups during the programme)	President’s Office, Regional Administration and Local Government (PO-RALG)	President’s Office, Regional Administration and Local Government (PO-RALG)
Regulator	Ministry of Health (MOH)	Ministry of Health (MOH)
Purchaser	National Health Insurance Fund (NHIF)	Ministry of Finance (MOF)
Fund holder	Ministry of Finance and Planning (MOF)	Ministry of Finance (MOF)
Verifiers	Internal verifier-Internal Auditor General (IAG) and a team identified by RAS (quarterly). External verifier -Controller Auditor General (annually)	No explicit verifier, but undertakes general annual audits across sectors including health by the Controller & Auditor General
Scale of implementation	9 regions (Mwanza, Pwani, Shinyanga, Geita, Kigoma, Kagera, Mara, Simiyu and Tabora)	All 26 regions in mainland Tanzania
Who received incentives/payment	Health workers and facilities, community health workers, health managers (CHMT and RHMT), and zonal MSD offices	All public health facilities in mainland Tanzania. Facility spending guide directs how funds should be used. There is 10% earmarked for staff motivation
Procedures in place to support facilities	Active Facility bank Account as per treasury guidelines Computerisation of HMIS Structural quality improvement since only facility with at least one star engaged in RBF 10 million TZS as start-up funds to improve facility infrastructure	Active Facility bank Account as per treasury guidelines Accountability mechanisms (FFARS and PlanRep) Deployment of accountants in health centres to support surrounding dispensaries. RHMT and CHMT supportive supervision

CHMT, Council Health Management Team; FFARS, Facility financial accounting and reporting system; HBF, Health Basket Fund; DHFF, Direct Health Facility Financing; HMIS, Health Management Information System; IAG, Internal Auditor General; MSD, Medical Stores Department; MOH, Ministry of Health; MOF, Ministry of Finance; NHIF, National Health Insurance Fund; P4P, Pay for performance; RAS, Regional administrative secretary; PlanRep, Planning and reporting system; PO-RALG, President’s Office, Regional Administration and Local Government; RHMTs, Regional Health Management Teams; RBF, Result Based Financing.

### 2.4 Study design

We adopted a mixed qualitative study approach, which includes document reviews as well as in depth interviews (IDI’s) and key informant interviews (KIIs) with key stakeholders knowledgeable about both donor funding schemes (RBF and DHFF-HBF) specifically on financial budgeting and spending. The approach involving data from multiple sources was considered appropriate for exploring a complex phenomenon in a real-life situation.

### 2.5 Conception framework

The study utilized the SHP framework developed by SPARC (Strategic Purchasing Africa Resource Center) to guide the data collection and analysis process (38). The framework was employed to describe each financial scheme under examination and explore how the configuration of purchasing functions influenced or restricted financial autonomy. The IDI’s and KIIs were specifically guided by four key purchasing functions (Table 2) (9, 39, 40): (1) the benefit specification (what to buy), (2) selective contracting (where to buy), (3) provider payment (how to buy), and (4) monitoring provider performance (Table 2). These purchasing functions were part of the SHP progress mapping framework co-developed by the SPARC and technical partners (41).

**Table 2:** Strategic health purchasing functions.

No.	Purchasing functions	Description
1.	Benefits specification	Specifying covered services and medicines and where they can be accessed. Cost-sharing policies and service delivery standards
2.	Contracting	Selecting public and/or private providers to deliver services in the benefit package and entering into contracts with them, specifying the terms and conditions in the contracts and enforcing the contracts
3.	Provider payment method	Selecting, designing, and implementing provider payment systems and setting payment rates
4.	Performance monitoring and accountability measures	Assessing provider performance, providing feedback for improvement, and carrying out system-level analysis of utilization, quality, and so forth to inform purchasing decisions

Source: SPARC framework on the ideal of strategic health purchasing functions (SPARC 2020).

Data collection also focused on how the purchaser-provider split is implemented within each financing scheme and the effect of each scheme on financial autonomy. The purchaser-provider split refers to the separation of functions between the purchasers and providers. It influences accountability and transparency in the allocation and utilization of funds. Financial autonomy focuses on the provider’s autonomy and responsibility in receiving, managing, and accounting for funds in the delivery of health services. Greater financial autonomy can enable providers to better match the payment to prioritized services (10).

### 2.6 Data collection

Data were collected through document review, IDIs and KIIs.

#### 2.6.1 Document review

We extracted information from various documents, with a focus on capturing information on the country’s strategic purchasing arrangements and functions under the two-donor funding schemes (RBF and DHFF-HBF). Documents were selected based on content accuracy in relation to strategic purchasing functions, accessibility, and policy relevance. Some of the documents that were reviewed include: the RBF design and operation manual, DHFF-HBF concept note and roadmap, policy documents, and RBF evaluation reports. We also reviewed published documents specifically on purchasing arrangements and functions of RBF and DHFF schemes in Tanzania.

#### 2.6.2 Key informant and in-depth interviews

We conducted face-to-face IDIs (*n* = 29) with various health stakeholders (Table 3), including representatives from PHC providers, district and regional levels in the Mwanza region. We chose stakeholders from one region, Mwanza, which was implementing both schemes. We also conducted (*n* = 2) KII’s with national-level stakeholders at the Ministry of Health level. Respondents were purposively selected based on their experience in donor funding programs, particularly experience with financial spending and budgeting at the PHC level as well as contracting and payment arrangements, RBF or DHFF scheme monitoring and implementation, or overall responsibility for health service delivery (e.g., District Medical Officers or RBF coordinators). The interview guides for IDI’s and KIIs were developed in English and translated into the local language (Swahili). Interviews were conducted in either language depending on the participants’ choice from February to March 2020. Interviews were audio recorded with the permission of the study participants. In order to minimize potential bias and subjectivity in this study and enhance the validity and credibility of the research findings, the interviewers were well-trained to conduct interviews with health stakeholders in a neutral and non-biased manner.

**Table 3:** Description of study stakeholders.

Targeted stakeholders	Number
**National stakeholders**	
Global Fund & RBF Coordinator (PORALG & MoH)	2
**Regional and district level stakeholders**	
Regional Medical Officers (RMO)	1
District Medical Officer (DMO)	1
District DHFF-HBF Coordinator	1
RBF Coordinator	1
**Healthcare providers**	
Providers in healthcare facilities (per district within Mwanza region)	25
**Total**	**31**

### 2.7 Data analysis

The study employed data triangulation to enhance its robustness comparing data from different sources related to two health financing schemes. We first synthesized information extracted from various documents. Audio-recorded data from IDI’s and KIIs were transcribed verbatim, and researchers reviewed the transcripts to familiarize themselves with the data. To further ensure the quality and credibility of the findings, the study underwent a peer review and external validation process. Health financing expert were invited to provide input and validation. Thematic content analysis was employed, involving both deductive and inductive coding, using NVivo version 12. This systematic and transparent data analysis approach involved multiple researchers independently coding and interpreting data to reduce subjectivity. Initial coding of the transcripts was carried out separately by experienced researchers, with input from other co-authors. Any disagreements were discussed to ensure consensus, and standards were set to guide the rest of the coding process. Similar codes were grouped into categories, and then themes were identified that were revised as new codes and categories emerged through the process. Our analysis used the four purchasing functions presented on Table 2 as themes, as well as purchaser-provider split, and financial autonomy to describe and compare each of the financing schemes.

### 2.8 Ethical considerations

The study was granted ethical approvals from national and institutional ethics committees in Tanzania. The institutional ethical approval was given by the Ifakara Health Institute (IHI/IRB/No: 003-2016). While the national approval was provided by the Ethical Committee at the National Institute of Medical Research (NIMR/HQ/R.8a/Vol. IX/2256). Health stakeholders who took part in the study were provided with an information sheet, which was explained further by the interviewers. Subsequently, informed consent was obtained with facilitators clearly explaining the study to the participants and securing their voluntary consent to participate. Moreover, the study ensured effective confidentiality during data collection by assuring participants that their identities would remain confidential, and audio recordings of interviews would be deleted once interviews were transcribed and gave consent for the anonymous use of quotes from interviews.

## 3 Results

Table 4 summaries the comparison between RBF and DHFF-HBF schemes across each of the SHP elements as well as the purchaser-provider split and autonomy. It provides a broad picture of how each funding mechanism differs and some similarities in relation to SHP functions as well as provider split and autonomy.

**Table 4:** Purchasing arrangement, provider splits and autonomy of DHFF-HBF and RBF schemes.

	RBF	DHFF-HBF	Differences	Similarities
**(a) Purchasing functions**				
**Benefits specification**	Facility level: 17 quantity indicators (include 3 indicators specific for CHW) on service utilization 14 quantitative indicators for dispensaries, health centres, and hospitals (Appendix A): new outpatient consultations, TASAF beneficiaries receiving outpatient’s care, children under one year immunized against measles, under-five receiving Vitamin A, new users of modern family planning methods, pregnant women receiving 2+ doses antimalarial, HIV-positive pregnant women receiving ARVs, mothers receiving post-natal care services within 3–7 days after delivery, pregnant women receiving ANC at least four times, HIV-exposed infants receiving ARVs, institutional deliveries, clients receiving HIV counselling and testing, TB case suspect referred and first antenatal visits 18 groups of indicators on service quality (including availability of essential health commodities).	Facility level: 6 quantity indicators of service utilization including Outpatient, Antenatal attendance, Institutional Deliveries, Postnatal attendances, Admissions, and C-sections 2 performance/quality indicators (availability of 30 tracer medicines and family planning use)	RBF purchased more quantity and quality indicators than DHFF-HBF	Focus on both quantity and performance/quality indicators at the facility level
**Contracting**	Purchasing services from health facilities, CHW, and district managers Provider types include public providers and private health facilities, as well as FBO’s (service agreement) Performance contract between providers and purchasers Specific contract verification	Purchasing services from health facilities, however, there are not explicit individual performance agreements with facilities. District health managers act as representatives of providers (public providers and private health facilities) entering into contracts for the delivery of agreed health services District health managers enter into contracts to oversee healthcare providers in achieving performance agreements	HBF does not contract CHW and individual performance agreements with facilities. HBF purchaser enters into a contract with the district manager to ensure oversight of the providers in implementing agreed performance goals. HBF has no specific contract for verification	Both entering into a performance contract
**Provider payment method**	Payment method: Fee-for-service adjusted with quality score Payment frequency: quarterly after data verification in all contracted providers	**Payment method:** Formula-based Capitation with adjustors for distance (10% for equity), catchment population (40% for need), outpatient, C-section, deliveries (40% for utilization) and availability of 10 tracer medicine and use of family planning (10% performance) for performance on six indicators, family planning use, and availability of 10 tracer medicines. **Payment frequency:** Quarterly. No explicit verification processes. Centrally validated HMIS data is used for adjustors.	RBF used fee-for-service, but HBF used capitation Differences in types and sizes of payment adjustors Determination of RBF payments and disbursement done on a quarterly basis, but HBF determination of payments done annually while disbursements are done quarterly	Both use of performance/ output-based payment methods The use of payment adjustors
**Performance monitoring and accountability measures**	**General supportive supervision:** Done by district health managers quarterly **Verification:** Done on a quarterly basis by internal and external verifiers (CAG), mostly in all contracted providers **Accountability measures:** Overall oversight at facility level done by HFGC, hospital boards, and health managers (CHMT, RHMT). Delayed use of public financial management (PFM) systems (particularly FFARs)	**General supportive supervision:** Done by health managers (CHMT and RHMT) quarterly **Verification**: No explicit verification process but CAG conducts annual financial and performance audit **Accountability measures:** Overall oversight at facility level done by HFGC, hospital boards, and health managers (CHMT, RHMT). The use of public financial management (PFM) systems (Plan-Rep and FFARs)	Advanced use of PFM systems in HBF than in RBF No explicit verification for DHFF-HBF	Both rely on the supportive supervision conducted by CHMT and RHMT and financial audit conducted by CAG Both use accountability measures
**(b) Purchaser provider split**				
	**Purchaser:** NHIF-responsible to purchase health services based on the predefined indicators from providers, but disbursement done by fund holder (Ministry of Finance and Planning (MoF)) **Provider:** Contracted health facilities, CHWs and health managers	**Purchaser:** MoH and PORALG **Fund holder:** MoF, pays the provider based on recommendations from the purchaser Payment adjustors and ceiling: MoH and PORALG are responsible for establishing and revise payment adjustors and use adjustors to determine payment ceilings for each facility on annual basis **Provider:** Contracted health facilities	Different types of purchasers: NHIF for RBF and MoH and PORALG for HBF There is no clear purchaser-provider split but at least payments are determined explicitly	Fund holder for both schemes MoF Both payments are determined explicitly
**(c) Autonomy**				
Budgeting process	Budgeting and development of plans done together with HFGC and guided by efforts to address specific needs at the facility (i.e., “Bottom-up and Need based”)	Budgeting and development of plans under the guidance of CHMT using the HF planning guidelines and Plan-Rep system	Budgeting and development of plans done together with HFGC under RBF, but guided by CHMT through guidelines for HBF	Both developed business plan and budgeting
Spending process	RBF not fully integrated to FFARS to maintain spending flexibility Spending criteria were based on the business plan and priorities at the time of spending -Priority on drugs and supplies, facility infrastructure based on facility priorities and needs, and related to RBF-incentivized service	Spending needs to be in alignment with approved facility plan and budget. Expenditure/procurement is guided by facility spending guideline and managed through FFARS Spending categories include: 35%_health commodities, 15%_Strengthen Human Resources for Health Capacity 45%_RMNCAH 5%_Community health system	More flexibility in spending RBF money compared to the budget ceiling for HBF RBF has full management and financial autonomy DHFF has limited management autonomy but full autonomy for planning, budgeting, and spending.	Both prioritized health commodities and improvement in service coverage and quality

CHWs, Community Health Workers; CHMT, Council Health Management Team; FFARS, Facility financial accounting and reporting system; FBOs, Faith-based Organizations; HBF, Health Basket Fund; HMIS, Health Management Information System; HFGCs, Health facility governing committees; MoH, Ministry of Health; MoF, Ministry of Finance and Planning; NHIF, National Health Insurance Fund; PlanRep, Planning and reporting system; PFM, Public financial management; PO-RALG, President’s Office, Regional Administration and Local Government; RHMTs, Regional Health Management Teams; RMNCAH, Reproductive, Maternal, Newborn, Child, and Adolescent Health.

### 3.1 Benefit specification

All public and private health facilities in Tanzania provide the package of services as directed by the Ministry of Health (MOH) to all Tanzanians. However, DHFF-HBF and RBF schemes incentivize performance on selected indicators. The DHFF-HBF scheme paid for fewer performance indicators compared to the RBF scheme (8 vs. 35). Both schemes include service utilization and quality of care indicators of performance. For the DHFF-HBF scheme, only two indicators are related to service quality. The quality indicators focus on the stock availability of 30 tracer or essential medicines, medical supplies, laboratory reagents, and vaccines. The RBF scheme had 18 groups of quality indicators which are purchased from PHC primary healthcare facilities (dispensaries, health centers, and hospitals). For both schemes’ quantity indicators are routinely measured by the existing HMIS, throughout the country. The RBF scheme quality indicators are assessed using a quality checklist and then incorporated into District Health Information System2 (DHIS2), while quality indicators for the DHFF-HBF scheme are directly extracted from the DHIS2 (each of the two indicators weighs 0.5) (37). The RBF scheme sets a fee for each service provided, while the DHFF-HBF scheme determines the weight of each unit, which is more generous in its approach.

### 3.2 Contractual arrangements

The central and local governments have the mandate to form a partnership and contracts with private providers and other funders to improve the delivery of health services. These partnerships are governed by soft tools such as memorandums of understanding rather than explicit contracts. Both RBF and DHFF-HBF schemes used contractual arrangements with district health managers/public PHC facilities for service provision. In some situations, both financing schemes also enter into a service agreement with non-public PHC facilities that have already been contracted by the government through a service agreement. However, there is a difference in how these agreements are structured; RBF use separate service agreement, while DHFF-HBF operates through the existing agreement established by the government.

In contrast to the DHFF-HBF scheme, the RBF scheme uses performance agreement (which defines each service indicator and how it will be achieved, and its fee). NHIF accredited the performance agreement to the public PHC facilities to provide specified health and management services of a specified quality. It also contracted additional agents such as CHWs and health managers. These agents such as CHWs were incentivized to improve the escort of women for delivery, household visits and reporting perinatal deaths, while health managers were incentivized on management indicators and overseeing surrounding facilities (21). On the other hand, under the DHFF-HBF scheme, the PORALG through Regional Administration, and Local Government Authorities (LGA’s), enters into a contractual agreement with all the District councils in Tanzania mainland. The agreement aims to provide oversight for public health facilities, ensuring the delivery of healthcare services to all citizens. There are no explicit individual performance agreements with facilities under the DHFF-HBF scheme.

Unlike the DHFF-HBF scheme, the RBF scheme has a specific contract for verification. The NHIF enters into a contract agreement with both external and internal verifiers. These verifiers include actors at the national (MOH, PORALG, and MOF), regional (RS and RHMT), and district levels (CHMT and DMO). The contract aims to facilitate, regulate, supervise and verify RBF scheme implementation. The contract specifies the responsibilities of each key actor in the RBF system. The MOF disburses the funds to the service providers in accordance with the RBF scheme contract and agreement. The RBF scheme also incentivizes CHWs and district health managers to perform specific indicators, which is not the case for the DHFF-HBF scheme.

### 3.3 Provider payment mechanism

Both schemes utilize output-based payment but differs in their provider payment method. For instance, the RBF scheme used fee-for-service (FFS) adjusted with quality scores to reimburse healthcare providers for providing healthcare services. The fee is the amount of money paid after providing service as pre-defined incentivized indicators. The payment is tied to pre-defined incentivized indicators that measure performance and quality. In the RBF scheme, 100% of the payment is based on performance across these indicators. Health providers are paid after data verification across all facilities on a quarterly basis.

The DHFF-HBF scheme uses a formula-based capitation model to pay health providers. The payment is done at a fixed rate per population served. The capitation allocation formula considers three adjustors; 40% for service utilization indicators, 10% for facility performance (quality indicators), 40% for population size (need), and 10% for distance from a district headquarters to a facility (equity). The performance comprises only 50% of the total payment. DHFF-HBF allocation per facility is determined annually, but actual disbursement is done quarterly.

### 3.4 Performance monitoring and accountability measures

In the DHFF-HBF scheme, there is no specific monitoring and accountability structure for HBF but rather general supervision and financial audit. In contrast, the RBF scheme incorporates verification in addition to general supervision and financial audit to monitor and ensure the accountability of providers. Both schemes rely on supportive supervision conducted by health managers (CHMT&RHMT), aiming to ensure effective monitoring of providers. The district and regional managers conduct quarterly supervision of health facilities. During the supervision, they assess facility’s operation and health indicators, which involves reviewing the facility’s performance, quality of care provided, adherence to protocols and guidelines, and overall implementation of the schemes. District health managers also provide coaching and guidance to address any identified gaps in order to ensure effective delivery of health services in accordance with the program’s requirements.

DHFF-HBF scheme also incorporates financial auditing as an approach to ensure providers compliance with financial procedures and audits. The financial auditing is conducted by the Controller Auditor General (CAG) annually, it involves selecting representatives from the providers as well as the health managerial level district and regional level. The providers are responsible for providing financial reports and implementing recommendations from auditors. Also, they are responsible for program reporting including monitoring results on health indicators and feeding data into national health statistics. For instance, in the DHFF-HBF scheme, providers are required to submit their financial reports annually at their respective district managers (typically at the DMO office) for auditing purposes.

The RBF scheme also uses verification as a way to monitor the performance of the healthcare facilities and ensure the accuracy of reported results. Internal verification is conducted by the internal auditor general in collaboration with regional secretariat using a monitoring checklist to verify results reported by providers on a quarterly basis before actual payments. External verification is conducted by Controller and Auditor General (CAG) to verify 25% of results from the internal verification, which is done annually. The performance monitoring of service provision is done through a contract (between the purchaser and provider) and the clinical quality checklist. The verification process helps ensure the accuracy of reported data and verify whether the providers are meeting the defined standards. These data form the basis for target follow-up actions to understand reasons for limited improvements.

In the initial phase of RBF implementation, providers had more autonomy in budgeting, spending, and reporting. However, there has been a shift towards standardization and alignment with the government financial management and reporting system. Both schemes currently use FFARS to ensure accountability among providers. The system is used to record budget disbursement, expenditure, and generates reports at the facility, council, regional, and national levels.

### 3.5 Purchaser-provider split

Unlike to the DHFF-HBF scheme, the RBF scheme implements a purchaser-provider split as a governance mechanism to enhance accountability and transparency in the allocation and utilization of funds. The purchaser of services for the RBF scheme was the NHIF. It has the responsibility of directing the MoF as to which services to buy from providers (health workers and managers). Moreover, NHIF has the mandate to sign contracts with PHC facilities and also to participate in the verification process, and approve payments after receiving the verification report (31). The RBF scheme targeted PHC providers (dispensaries, health centers, and district hospitals). Apart from providing health care, providers also prepared reports including HMIS reports, financing, and technical reports of the business plans.

The DHFF-HBF scheme does not have a clear purchaser-provider split, but the payment arrangements are explicitly determined. The MoH and PORALG act as the purchaser of the service. They work collaboratively to define the benefit package to be purchased, establish and revise payment adjustors and determine payment ceilings for each facility on an annual basis through HBF. The MoF serves as the fund holder and payer, receiving calculated payments from MoH and PORALG and disbursing funds to facilities accordingly. The service providers are all primary healthcare facilities in mainland Tanzania. They also set priorities and procure a mix of inputs to produce and deliver health services to clients and the community. The providers have the function of preparing planning, budgeting, procuring, accounting, reporting, human resource management, as well as monitoring and evaluation (10, 33).

### 3.6 Financial autonomy

Budgeting and spending decisions within RBF scheme have been “bottom-up” and “needs-based,” while DHFF-HBF scheme budgeting and spending decisions were largely “top-down” and “rules-based.” There were systematic differences in how budgeting and spending decisions were approached in the initial phase of RBF scheme, and how such decisions are approached after the implementation of DHFF-HBF scheme. While RBF scheme budgeting and spending initially were guided by efforts to address specific needs at the facility, decisions under DHFF-HBF scheme were largely following guidelines and protocols.

*“The 75% of the funds which the health facility receives from RBF depends on the needs of the health facilities, for example when RBF started many facilities did not have incinerators or placenta pits. So the priority was on incinerators and placenta pits and they completed that. The other priority which they did is that many facilities did not have benches for patients to sit while waiting for services, notice boards, suggestion boxes so they bought all those. They also prioritized improvement in water supply and electricity, buying sim-tanks for harvesting rain water and installed electricity and solar power. The 75% of funds also were used to buy drugs in ensuring availability of medicine.”* (Health Secretary, Misungwi district, Mwanza)

And

*“it (RBF) has given providers a wide choice of spending the 75% particularly on items related to performance indicators or overall facility improvement.”* (Region Medical Officer, Mwanza)

However, since 2018/2019 the budgeting and spending categories are pre-determined in the electronic financial systems (PlanRep and FFARS) for both DHFF-HBF- and RBF funds. This has reduced flexibility in RBF scheme spending. Mainly because the budgeting and spending categories are pre-determined and follow a predetermined structure which may not always align with a facility’s strategic priorities. It is also challenging to address unexpected needs at the facility:

*“Initially on RBF we used to spend according to our needs when we receive money, but now we are following the approved budget. If you planned to do something then you no longer have to enter something else, you must follow the budget that was approved.”* (Facility in-charge, Sengerema district council, Mwanza)

Restrictions on how HBF-DHFF funds can be budgeted and spent were seen as a major constraint by a majority of health providers. For instance, some health providers were frustrated that the DHFF-HBF scheme could not be budgeted to pay for construction work as well as expenses such as allowances to doctors on call and payment of casual laborers. They asked for greater autonomy in deciding how to spend money and respond to unexpected challenges that were not foreseen in the annual plans, and they complained about the limited flexibility to change items in response to changing needs.

*“(…) sometimes the money is there but there is something you have to prioritize but because it was not on the budget during planning then you may not implement it […]”.* (Facility in-charge, Kwimba district, Mwanza)

*“There is an action plan and everything is ready, they have already [been] allocated; if 50% is supposed to go to medicine then it should go there, if it is for hospital supply it should go there. I mean you cannot take money here and use it for something else such as construction…”* (Facility in-charge, Sengerema district council, Mwanza)

The RBF funds were meant to be spent as follows: 75% for facility improvement and 25% as staff incentives. Most health providers reported to have spent much of their 75% RBF funds for the purpose of improving working conditions and quality of care, while increased service utilization has received significantly lower priority.

*“RBF money which has been deposited now…there is 25% incentives for staff then there is 75% for improving service at the respective facility. For example, we have planned to use that 75% of RBF money to build house for staff because we only have one house for the in-charge, others we are renting houses in the community, therefore we have proposed to build one house to accommodate two staff.”* (Facility incharge, Magu district, Mwanza)

In particular, facilities used RBF funds to procure drugs and supplies and improve facility infrastructure based on facility priorities and needs. Priority was generally given to medicines that related to the delivery of RBF-incentivized services (e.g., provision of anti-malarial drugs and iron tablets). Investments were also commonly reported in facility infrastructure, including procurement of furniture (e.g., chairs, tables, benches, and door handles).

DHFF-HBF scheme-spending covered similar areas to RBF scheme but was more heavily and consistently focused on procuring medicines. This is partly because the DHFF-HBF guidelines required each facility to spend one-third of HBF on drugs. Other areas of investment included the procurement of gas tanks and gas refills to store vaccines, funding for emergency transport for safe referral for delivery, and airtime for staff. DHFF-HBF-funds were also used to pay per diems to health facility governing committee members to attend meetings.

*“Most of the basket money goes to drug supply, this is the first priority so most of the money goes there. Drug supply, and another area is action plan when the budget is brought at the end of the year.”* (Facility in-charge, Sengerema district, Mwanza)

Facility spending on various priorities used funds from both RBF and HBF in a complementary way. In the presence of inadequate funding, one source of fund would complement the other.

*“(…) there is no way you can say RBF money can cover everything, it has never happened, to be honest it is because RBF money finishes, and you take some out from the Basket Fund, that’s why I say in general they depend on each other*.” (Facility in-charge, Kwimba district, Mwanza)

## 4 Discussion

This is the first study to describe the SHP arrangements, purchaser providers split, and degree of financial autonomy between the DHFF-HBF and RBF schemes in Tanzania. Overall, we found purchasing arrangements of both schemes were largely aligned with SHP principles. For example, both schemes clearly defined their purchasing arrangements that specified what to purchase, how they should be purchased and from whom. Moreover, both schemes prioritize services through explicit benefit packages, contracting with both public providers and in some cases private providers, paying health providers through capitation and fee for service methods, and various forms of provider performance monitoring and accountability mechanisms including supervision and auditing as well as the government’s financial management systems. While the RBF scheme fully implements a provider-purchaser split, the DHFF-HBF scheme incorporates elements of such a split in its implementation. Both schemes include ways to strengthen autonomy.

This study also revealed some discrepancies in purchasing arrangements between the two schemes. For example, in benefit specification both schemes used output-based payment methods which enable health purchasers to “buy the right thing” and better match payment to prioritized services (2). However, RBF scheme has more of a performance-based payment involving a larger set of explicit performance indicators and incentivizes providers based on those indicators, while DHFF-HBF scheme includes partial performance-based payments using fewer performance indicators, although the inclusion of outpatient visits provide space to accommodate more services. The discrepancy in the number of performance indicators between schemes may be attributed to differing goals underlying each scheme: DHFF-HBF’s primary focus on ensuring adequate health resources reaching the frontline providers as highlighted in another Tanzania study (35). Whereas RBF program focuses on linking financing to results to achieve health targets as well as to motivate frontline providers. Both programs aim to mobilize and empower frontline providers to improve coverage, quality and accessibility to health services in order to achieve UHC (23).

In LMICs, governments heavily rely on donor funding for health services (42). This reliance has led to a high degree of fragmentation in health financing, characterized by multiple funding mechanisms (43, 44). In Tanzania, Global Fund and GAVI through vertical programs covers services like HIV, TB, Malaria and immunization programs (45), which are partially funded domestically and by other donors. This lead to duplication of the services purchased or funded, programs are more expensive and designed to merely provide data, increased burden in reporting among providers, and inequities in access to affordable healthcare (42). Through the HBF and a recent introduction of DHFF, the Government of Tanzania has encouraged donors and development partners to align their funding in one pool of HBF, aiming for a higher level of pooling and harmonized provider payment method (34, 46).

The DHFF reform gives a platform to fund PHC facilities directly countrywide, using funds from multiple sources. RBF and DHFF-HBF both focused on paying direct public PHC providers based on pre-defined indicators (contractual agreement), with few contracted non-public PHC facilities based on service agreement. This approach of expanding contractual arrangement helps to address gaps in healthcare provision and supports the goal of providing accessible services to the entire population (47). Unlike for RBF scheme which has performance agreement, the DHFF-HBF scheme had no explicit performance contract between the purchaser and each provider, but relied on district-level agreements focused on oversight. This may weaken the link between performance and payment, limit the provider accountability, inequitable resource allocation, flexibility in service delivery, and reduce autonomy (48). The RBF scheme incentived multiple health system agents, beyond providers, such as CHWs and health managers; who late created a mutual dependency with health facilities and improved their cooperation and trust and facilitated increased utilization of health services (21). HFGCs are key health system agents in overseeing health facilities and financial accountability, but were not paid by RBF nor DHFF schemes.

RBF and DHFF-HBF schemes use payment adjustors to ensure efficiency in resource allocation. RBF scheme used a fee-for-service adjusted by quality score, while the DHFF-HBF scheme uses capitation adjusted by distance, catchment population, and service utilization. These payment methods focused on output-based payments, which aligns well with strategic healthcare purchasing. The output-based payments enhance equity, incentivize continuous improvements and innovations, galvanize trust in the community, and ultimately, foster increased service utilization (23, 49). The RBF scheme in Tanzania did not include remoteness incentives, unlike some other countries that have implemented RBF schemes. In Zimbabwe, for instance, the RBF scheme included payments specifically designed to address the challenges of remoteness (23, 48). A recent study in Tanzania reported that in some regions, 69% of the population lives relatively far from a health facility (23). In response, the DHFF-HBF scheme includes distance as a payment adjustor to account for barriers to health service access (46). Geographical or equity targets involve providing high incentive bonuses to providers serving disadvantaged clients or remote populations (15, 50). When comparing the two schemes, it is noted that they generally complement each other, with DHFF-HBF serving as a base allocation to all facilities without overly restrictive performance measures, while the RBF scheme acts as an additional motivator for facility performance.

Despite the potential for payment based on output, each method has its own effects on influencing healthcare provider behavior (intended or unintended) and affect healthcare delivery (51). For instance, fee for service may influence overprovision of services and prioritize profitable treatments over preventable care (52). One study reported that reimbursement by capitation systems was associated with a 22% lower cost compared with fee for service systems (53). On the other hand, capitation may influence under-provision of care rather than optimizing it for their patients, for example it may lead avoiding enrollment of unhealth patients (54). Thus, many country combine payment methods to create a blended payments system or mixed model, which can positively influence providers, leading to improved service quality and cost-effectiveness because they complement each other (52, 55).

In both DHFF-HBF and RBF schemes, payments were made on a quarterly basis, although their structure differed: in the RBF scheme, payments were made after verification in all contracted providers. On the other hand, DHFF-HBF scheme, allocation per facility is determined annually, but actual disbursement is done quarterly. Although shorter payment intervals may be associated with a higher administrative burden (56), it offers a stronger incentive by influencing positive provider behavior (57). However, the annual allocation and quarterly disbursement in the DHFF-HBF scheme, may impact providers ability to respond effectively to unexpected needs and urgent situations due to limited financial flexibility, incentive misalignment, and resource constraints.

We have found that there is a large variation in the provider monitoring and accountability measures between the two schemes. In the DHFF-HBF scheme, monitoring and accountability for the HBF rely on general supervision, without a specific structure or explicit verification process. While the RBF scheme goes beyond general supervision and financial audit by conducting data verification across all targeted facilities. RBF involves more comprehensive verification of performance scores at health facilities and community levels. Different countries have adopted alternative verification approaches for RBF. The approach used in Tanzania is similar to that in Burundi and Rwanda with a focus on error correction and learning, with a view to supporting health systems strengthening. While countries that were more focused on financial accountability, like Argentina, Afghanistan, and the United Kingdom, used it mostly for sanctioning or cost-recovery purposes (58). The reason for the DHFF-HBF scheme not to incorporate data verification may have been driven by cost and time constraints. Studies revealed that verification processes within RBF have been found to be complex, costly, and time-consuming (24, 59). In Benin for instance, a study found that the costs of the verification, and in particular the cost of the community verification, are high as compared to the RBF funds disbursed to the service providers (24). For example, verification activities made up 16% of the total costs of the national RBF program, and verification cost 25%–30% of the entire budget (59). Other studies have documented the delays in service provision and payment to the providers due to the difficulties associated with validation processes (60, 61).

We have found there is a significant variation in the purchaser-provider split between the RBF and DHFF-HBF schemes. In the RBF scheme, a clear and explicit provider-purchaser split is fully implemented, delineating the roles and responsibilities through contracts to each party in order to establish clear lines of accountability in planning, resource allocation, and monitoring performance. For the purchaser-provider split mode to be strategic, the operations of both entities should be managed by contracts (62). A study conducted in Tanzania reported that the approach allows for specialized focus and expertise in different aspects of the healthcare system, ultimately contributing to improved access to and quality of healthcare services for the population (47). However, in the DHFF-HBF scheme, where the separation is not fully established, the multiple roles are played by two entities; the MoH and PO-RALG. For instance, they act as regulators, facilitators, and purchaser of the service within the DHFF-HBF scheme. Studies reported that the absence of purchaser-provider splits limits the financial autonomy of providers and hinder their ability to respond to service delivery needs, resulting in failure to promote quality and efficiency of service delivery (63, 64).

Both schemes have strengthened autonomy, particularly in how budget and spending decisions were made. However, the RBF scheme was more flexible in budgeting and spending compared with the DHFF-HBF scheme. In the RBF scheme, 75% of funds were meant to be spent on facility improvement. Therefore, providers have more autonomy and flexibility in determining how to allocate and use the funds based on their specific needs and priorities. The decision under the DHFF-HBF scheme is highly guided by protocols and guidelines. In strengthening accountability to the providers both DHFF-HBF and RBF schemes manage their funds through FFARS (65), which increases efficiency and reduces system and administrative fragmentation across sectors and levels of government. However, while FFARS enhances accountability, it constrains autonomy in spending. The restriction to the use of FFARS for both schemes has reduced RBF flexibility to some extent. There is a need to increase autonomy in deciding how to spend money by reducing the budgeting and spending codes listed on the chart of accounts approved by the MoF. The mechanism should allow flexibility in the reallocation of funds within the approved budget. This will improve the provider’s ability to respond to unexpected challenges that were not foreseen in the annual plans (65).

Our study has important implications for both policy and research. Both schemes follow the principles of strategic purchasing. In this regard, our study highlights that strategic purchasing can take different forms each with strengths and weaknesses. It is high time for Tanzania policymakers to capitalize for strategic and effective design elements of each scheme. For instance, since the RBF has phased out, the DHFF design can be improved to accommodate lessons learned from the RBF implementation, such as enhanced autonomy in spending, close monitoring, and verification. More research into the effects of each on use and quality of care is needed. However, it is important to acknowledge that there are different approaches to strategic purchasing, and further evidence is needed to understand the advantages and disadvantages associated with each scheme.

The study acknowledges two main limitations. Firstly, both schemes have undergone further changes and redesigning since our interviews were conducted. However, informal interviews with government officials were conducted recently to gather additional information and ensure clarity on key issues. Secondly, there was a discrepancy in the level of awareness among healthcare providers regarding SHP functions compared to budget and spending issues. To address this gap, document review was utilized. It is crucial to consider these limitations and recognize that the schemes may have evolved since the study was conducted, requiring careful interpretation of the results and implications.

## 5 Conclusion

The implementation of SHP in two health financing programs (RBF and DHFF-HBF) in Tanzania demonstrates its high potential of ensuring efficiency in the allocation and spending of the pooled funds. The study findings support this notion by demonstrating the effectiveness of SHP in prioritizing services through explicit benefit packages, contracting with both public and private providers, paying health providers through output-based payment, and various forms of provider performance monitoring paving the way for more effective and sustainable healthcare financing systems. The government’s effort of defining a clear benefit package and entering into a contract with non-public health facilities helps to strengthen SHP functions hence improving access to services countrywide. The government’s commitment to Direct Facility Financing aims to provide the provider autonomy in budgeting and spending while enhancing provider accountability through systems like FFARS. However, it is important to review the use of PFM systems (for example FFARS) to increase provider autonomy in spending, enabling them to respond effectively to unexcepted changes in healthcare delivery. As some of the healthcare financing reforms are taking place in the country, purchasing functions should be reviewed to increase the possibility of accelerating the country’s progress toward UHC.

## Data availability statement

The original contributions presented in the study are included in the article/supplementary material, further inquiries can be directed to the corresponding authors.

## Ethics statement

The study was granted ethical approvals from national and institutional ethics committees in Tanzania. The institutional ethical approval was given by the Ifakara Health Institute (IHI/IRB/No: 003-2016). While the national approval was provided by the Ethical Committee at the National Institute of Medical Research (NIMR/HQ/R.8a/Vol.IX/2256). Health stakeholders who took part in the study were provided with an information sheet, which was explained further by the interviewers. The facilitators explained to the participants that their participation was fully voluntary. All participants were assured that audio recordings of interviews would be deleted once interviews were transcribed and gave consent for the anonymous use of quotes from interviews.

## Author contributions

JM: Conceptualization, Data curation, Formal analysis, Methodology, Software, Validation, Visualization, Writing – original draft. NS: Conceptualization, Writing – review & editing. RC: Writing – review & editing. GM: Data curation, Validation, Writing – review & editing. JB: Conceptualization, Data curation, Methodology, Visualization, Writing – review & editing. NK: Writing – review & editing. PB: Conceptualization, Methodology, Visualization, Writing – review & editing.

## Appendix A

Quantity indicators of RBF and DHFF-HBF schemes.

**Table:**

	RBF quantity indicators	Fee for each indicator	DHFF-HBF quantity indicators	Weight for each indicator
1.	No. of new outpatient consultations	($0.25)	Outpatient	01
2.	No. of low-income individuals identified by TASAF hybrid proxy testing receiving outpatient care	($0.75)	Antenatal attendance	01
3.	No. of children under one year immunized against measles	($5.00)	Institutional deliveries	06
4.	No. of under-five receiving Vitamin A supplementation	($3.75)	Postnatal attendances	01
5.	No. of new users on modern family planning methods	($0.75)	Admissions	10
6.	No. of pregnant women receiving 2+ doses of intermittent presumptive treatment of malaria	($2.00)	C-sections	27
7.	No. of HIV-positive pregnant women receiving ARVs	($12.50)		
8.	No. of mothers receiving post-natal care services within 3–7 days after delivery	($5.00)		
9.	No. of pregnant women attending for ANC at least four times during pregnancy	($1.00)		
10.	No HIV-exposed infants receiving ARVs	($0.50)		
11.	No of institutional deliveries	($3.50)		
12.	12. No of clients initiated by health care providers to counsel and test for HIV (PITC)	($0.37)		
13.	No of TB suspect referred (already screening)	($3.00)		
14.	No of first antenatal visits, with gestation age < 12 weeks (quantity)	($5.00)		

## Glossary

D by D: Decentralization by devolution
DHFF-HBF: Direct Health Facility Financing of Health Basket Funds (HBF)
DHIS2: District Health Information System2
EACs: East African Countries
GoT: Government of Tanzania
HSSF: Health Sector Service Fund
CHWs: Community Health Workers
CHMTs: Council Health Management Teams
KIIs: key informant interviews
LMICs: Low and Middle-Income Countries
MoF: Ministry of Finance and Plan
MoH: Ministry of Health
P4P: Pay for Performance
PFM: Public Finance Management
PO-RALG: President’s Office – Regional Administration and Local Government
PHC: Public Primary Healthcare
RBF: Result Based Financing
RS: Regional Secretariat
SHP: Strategic Health Purchasing
UHC: Universal Health Coverage
WHO: World Health Organization
